# An Account of an Extraordinary Conformation of the Heart

**Published:** 1786

**Authors:** Richard Pulteney


VII. *An Account of an extraordinary Conformation of the Heart. By* Richard Pulteney, *M. D. F. R. S.*—Vide *Medical Tranſactions publiſhed by the College of Phyſicians in London*, Vol. III. 8vo. London, 1785.

THE young gentleman whoſe caſe is here related had uniformly been ſubject to a

ſenſe

ſenſe of oppreſſion and faintneſs, as well as to difficulty of breathing, after any muſcular exertion; and for ſome time before his death theſe complaints gradually increaſed, ſo that, at length, he frequently could not walk acroſs a room without turning almoſt black in the face and hands, and becoming at the ſame time faint and almoſt breathleſs. He lived in this unhappy ſtate till the age of thirteen years and nine months, when he ſunk under a dyſentery, with which he was ſeized on coming off a journey.

On diſſection the adipoſe membrane of the external parts of the body was found to be extremely loaded with fat.

In the abdominal viſcera nothing particularly remarkable was obſerved, except that the gall bladder was nearly divided by a membranous ſtricture into two cavities.

Both the lobes of the lungs were found to be remarkably ſmall, and collapſed; and ſome parts of them flaccid to ſuch a degree, as to ſuggeſt the idea of their having been incapable of performing their function.

The heart was firm in its texture, and of a natural ſize, but the left auricle was obſerved to be very ſmall. No defect could be diſcovered vered in the tricuſpid or in the mitral valves of the heart; nor in the ſemilunar valves of the aorta, or in thoſe of the pulmonary artery: but on examining the ventricles and aorta, a canal was found communicating with both the ventricles. This was ſituated in an oblique direction, near the baſis of the heart, and was ſo capacious as to admit the end of a finger to be paſſed from the aorta, with equal facility, into either ventricle, the ſeptum of the ventricles appearing to terminate with this canal, which was totally unfurniſhed with any thing like valves, and was in every part perfectly ſmooth.

On examining the entrance of the pulmonary artery, within the ventricle, it was judged that, in this ſubject, the ring itſelf was much ſmaller, and more firm than is uſual.

From this ſtricture of the pulmonary artery, and the collapſed appearance of ſome parts of the lungs, the learned author thinks it probable that, at all times, a very inſufficient quantity of blood was circulated through the lungs; and when we add to this the facility with which the returning blood found its way through the canal, from the right to the left ventricle, it ſeems, he obſerves, neceſſarily to follow, follow, that, on any mufcular exertion, the heart muft have been overloaded; the difficulty of the circulation greatly increafed; and the blood detained in all the ramifications of the fanguineous fyftem. In this manner Dr. Pulteney accounts, in a fatisfactory manner, for the fymptoms under which the patient laboured, fuch as the fenfe of oppreffion upon motion; the faintnefs and difficulty of breathing; and the more or lefs conftantly-deepened tinge of the fkin, particularly that of the face and hands.

